# Genicular artEry embolizatioN in patiEnts with oSteoarthrItiS of the Knee (GENESIS) Using Permanent Microspheres: Interim Analysis

**DOI:** 10.1007/s00270-020-02764-3

**Published:** 2021-01-20

**Authors:** M. W. Little, M. Gibson, J. Briggs, A. Speirs, P. Yoong, T. Ariyanayagam, N. Davies, E. Tayton, S. Tavares, S. MacGill, C. McLaren, R. Harrison

**Affiliations:** 1 University Department of Radiology, Royal Berkshire NHS Foundation Trust, Reading, UK; 2 Department of Orthopaedics, Royal Berkshire NHS Foundation Trust, Reading, UK; 3 University of Reading, Reading, UK

**Keywords:** Osteoarthritis, Knee, Embolization, Genicular

## Abstract

**Purpose:**

Planned interim analysis of GENESIS; a prospective pilot study investigating the role of genicular artery embolization (GAE) in patients with mild to moderate osteoarthritis of the knee using permanent microspheres.

**Methods:**

Thirty-eight patients, median age = 60 (45–83), attended for GAE using 100–300 μm permanent microspheres. All patients had mild to moderate knee OA, resistant to conservative treatments over 6 months. Knee MRI was performed at baseline, and 12 months, enabling semi-quantitative analysis using Whole-Organ Magnetic Resonance Imaging Score (WORMS). Knee Injury and Osteoarthritis Outcome Score (KOOS) and visual analogue scale (VAS) (0–100 mm) were completed at baseline, 6 weeks, 3 months (*n* = 32), and 1-year (*n* = 16). Adverse events were recorded prospectively.

**Results:**

Technical success of accessing and embolizing the target genicular arteries was 84%. Six patients were not embolized: four due to a presumed risk of non-target embolization, and two due to a lack of hyperaemic target. Mean VAS improved from 60 (SD = 20, 95% CI 53–66) at baseline to 36 (SD = 24, 95% CI 28–44) at 3 months (*p* < 0.001) and 45 (SD = 30, 95% CI 30–60) at 1-year (*p* < 0.05). All KOOS subscales showed a significant improvement at 6-weeks, 3-months, and 1-year follow-up, except function in daily living, which reached borderline significance (*p* = 0.06) at 1-year. Four patients experienced mild self-limiting skin discoloration over the embolized territory. One patient experienced a small self-limiting groin haematoma. WORMS scores at 1-year follow-up showed significant improvement in synovitis (*p* < 0.05). There were no cases of osteonecrosis.

**Conclusion:**

GAE using permanent microspheres in patients with mild to moderate knee OA is safe, with potential efficacy at early follow-up.

## Introduction

Osteoarthritis (OA) of the knee joint is the most common articular disease of the developed world, and a leading cause of chronic disability, and economic burden [1]. Treatment options include analgesia, physiotherapy, intra-articular steroid, platelet-rich plasma (PRP), or hyaluronic injections, education programs, weight loss, and anti-inflammatory preparations. Joint replacement surgery is generally reserved for those with severe joint disease, pain, and functional limitation [2]. Despite this, expectations of surgery are not met in up to 30% of patients undergoing total knee replacement (TKR) [3]. Mild to moderate knee OA, not yet severe enough to warrant joint replacement, and resistant to nonsurgical options, represents a specific management challenge that justifies research into the area.

Like many multi-factorial conditions, there remains to be a grand unified theory to explain the pathogenesis of OA. Work has elucidated the role of angiogenesis in the pathophysiology of knee OA. Mapp et al. found that angiogenesis is increased in the synovium, osteophytes, and menisci [4]. New abnormal blood vessel growth results from macrophage activation within the synovium that is driven by inflammation. Pro-angiogenic factors such as vascular endothelial growth factor (VEGF), β-nerve growth factor, and neuropeptides have been identified as propagating neo-angiogenesis, and disrupting the osteochondral junction [5–7]. As sensory nerves grow along new blood vessels in osteoarthritic joints, they eventually penetrate non-calcified articular cartilage, osteophytes, and the inner regions of the menisci. It is hypothesized that angiogenesis contributes to structural damage and pain in OA, and thus may therefore provide a potential embolization target. Okuno et al. were the first group to describe their experience of genicular artery embolization (GAE) in patients with knee OA [8]. Since then, a small number of studies have reported on the role of GAE in the treatment of knee OA [8–12]. The Genicular artEry embolizatioN in patiEnts with oSteoarthrItiS of the knee (GENESIS) study investigates the safety and feasibility of performing GAE in patients with mild to moderate knee OA using permanent microspheres.

## Materials and Methods

GENESIS is a prospective single-centre pilot study with full ethical approval and is adopted onto the National Institute for Health Research (NIHR) portfolio (IRAS: 237676, CPMS: 37741). Planned interim analysis was performed to assess safety, and feasibility of GAE. Patients presenting to the department of orthopaedics with knee OA were considered for the study. Inclusion criteria included age 45 years or older, mild to moderate knee OA as determined on X-ray as Kellgren–Lawrence (KL) grade 1–3, and knee pain present for at least 6 months, resistant to conservative treatment (physiotherapy/analgesia/exercise/weight loss/intra-articular injections). Patients were excluded if they had rheumatoid or infectious arthritis, severe knee OA (KL-grade 4), renal impairment (eGFR < 45), a bleeding diathesis, irreversible coagulopathy, or previous knee arthroplasty. All patients were reviewed in clinic by a consultant orthopaedic surgeon and deemed suitable for inclusion. They were then assessed by the interventional radiology research team and consented for inclusion into the study.

Patients’ symptoms were evaluated using the Knee Injury and Osteoarthritis Outcome Score (KOOS) questionnaire, and a visual analogue scale (VAS) (0–100 mm) [13, 14]. These outcome measures were repeated at 6-week, 3-month (*n* = 32), and 1-year (*n* = 16) post-GAE. The KOOS questionnaire is a validated tool consisting of five subscales (pain, other symptoms, function in daily living, function in sport and recreation, and knee related quality of life) able to quantify changes in knee OA post-intervention; a normalized score (100 indicating no symptoms and 0 indicating extreme symptoms) is calculated for each subscale. Patient’s knee-specific analgesia use was recorded at baseline, 6-weeks, 3-months, and 1-year follow-up. Paracetamol, non-steroidal anti-inflammatory drugs (NSAIDs), and opiates were recorded. Finally, patient satisfaction questionnaires were designed to ascertain patient-reported outcome measures (PROM) on the GAE procedure. The questionnaires were completed by the patients prior to discharge on the day of the GAE procedure.

Prior to GAE, participants underwent contrast-enhanced MRI of the knee to allow non-invasive assessment of synovial hypervascularity as previously described [15, 16]. Images were acquired on a Philips Ingenia 3T MRI scanner. Proton density fat-saturation (PDFS), sagittal (Slice-thickness (ST) 2.5, TR 4177 ms, TE 30 ms, field of view (FOV) 144, matrix 360 × 286), axial (ST 3, TR 4589, TE 30, FOV 144, matrix 360 × 275), and coronal sequences (ST 2.5, TR 4848, TE 30, FOV 160, matrix 516 × 366) were acquired. In addition, contrast-enhanced imaging was acquired using axial T1-FS (ST 3, TR 578, TE 20, FOV 144, matrix 344 × 276), and sagittal T1-FS (ST 2.5, TR 697, TE 20, FOV 144, matrix 344 × 276) sequences. MRI of the knee was repeated at 12-month follow-up. Whole-Organ Magnetic Resonance Imaging Score (WORMS) was used to standardize imaging assessment pre and post-intervention as previously described [17]. Images were independently reviewed by two musculoskeletal radiologists with 11 and 6 years of experience. Both radiologists had prior training and experience of WORMS. They were blind to patient characteristics and outcome measures (KOOS and VAS).

### Genicular Artery Embolization for Knee OA

**Fig. 1:**
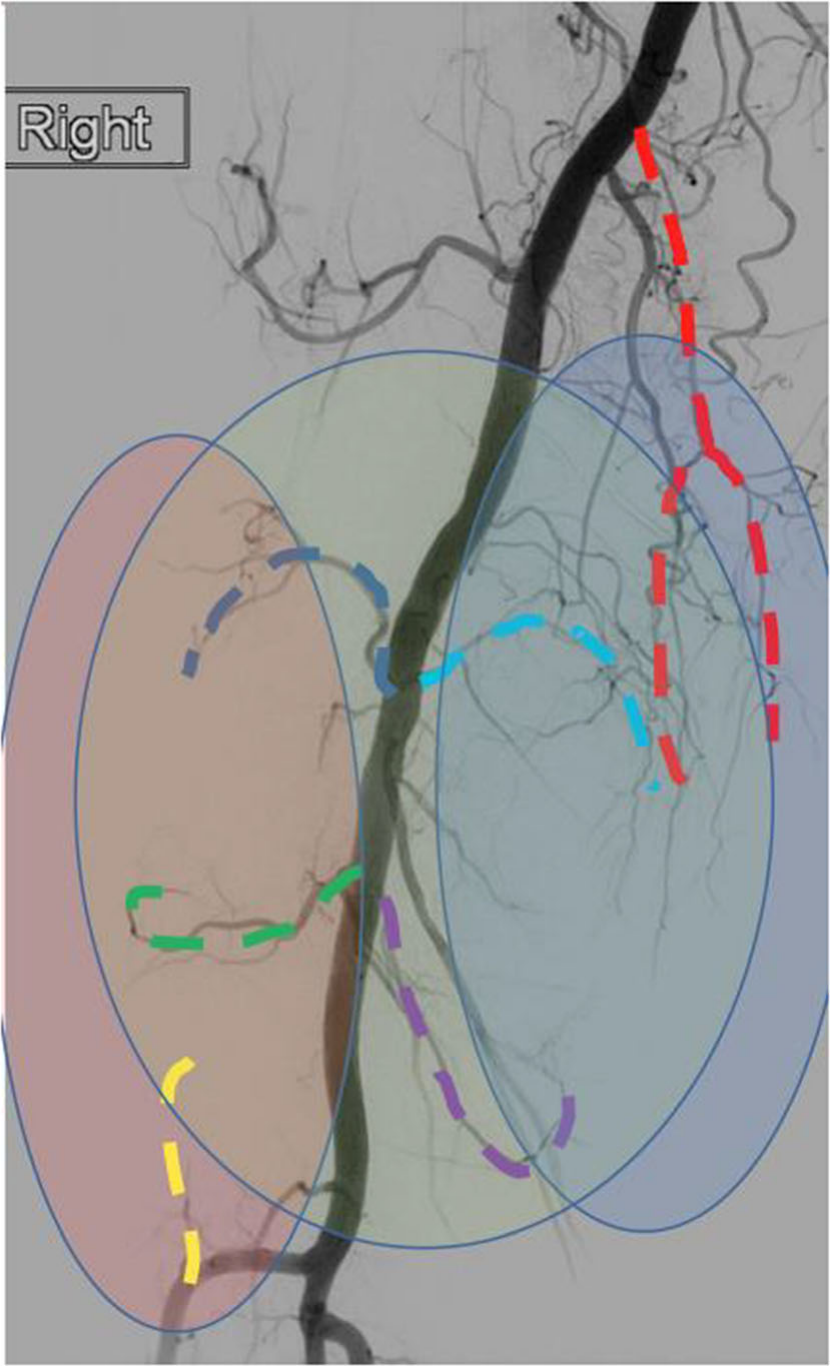
Non-selective angiography from the distal superficial femoral artery, revealing the genicular arterial anatomy. The medial joint compartment is supplied by the descending (red), superior (light blue), and inferior (purple) medial genicular arteries. The lateral joint compartment is supplied by the superior (dark blue) and inferior (green) genicular arteries, as well as the anterior tibial recurrent (yellow) genicular artery. The patellofemoral joint takes arterial supply from all six genicular arteries

**Fig. 2:**
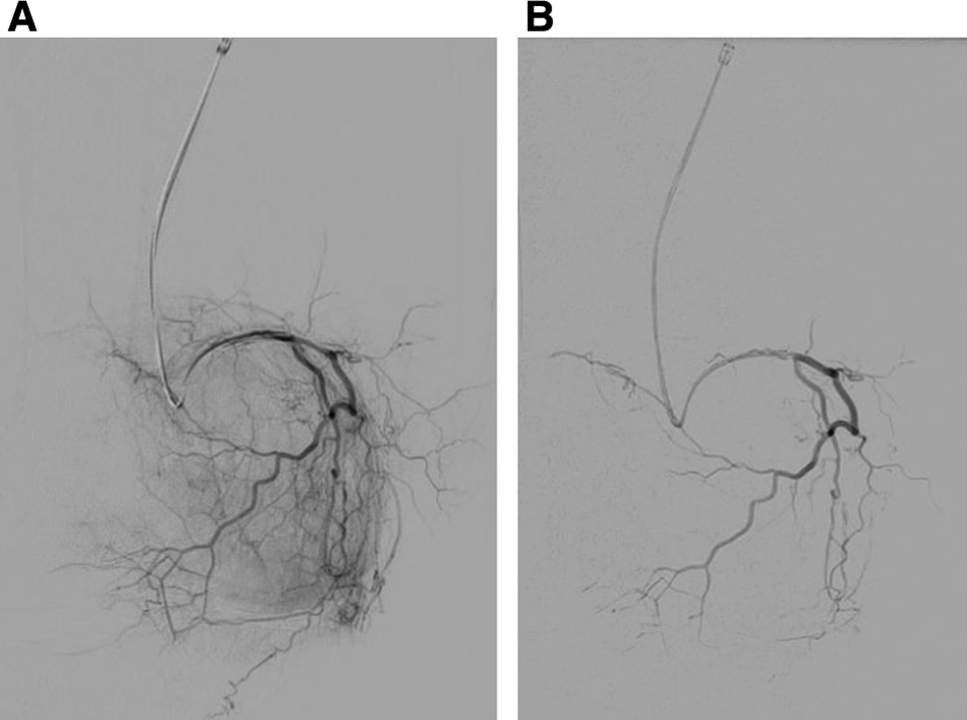
**A** Superior medial genicular artery is selected revealing hyperaemic synovium within the medial knee compartment in a patient with grade 2 KL knee OA. **B** Area was embolized with 2 ml 100–300 μm Embosphere particles diluted in 20 ml contrast, to prune the abnormal vascularity revealing the post-embolization appearance

All GAE procedures were carried out by two consultant interventional radiologists with 6 and 25 years’ experience. Ultrasound-guided anterograde access of the common femoral artery was performed, with insertion of a 4F vascular sheath (Cordis Medical, USA). A hydrophilic guidewire (Terumo, Japan) and Cobra Performa catheter (Merit Medical, USA) were used to gain access to the distal superficial femoral artery, from where angiography was carried out using 300 mg/ml iodinated contrast (Iomeron, Bracco, Italy) to visualize the genicular arterial anatomy (Fig. 1). A hyperaemic blush signified the pathological target as previously described [9, 10, 16]. Using a Fathom 14 guidewire (Boston Scientific, USA) for vessel selection, a straight-tip microcatheter (2.9–2.0F Pursue, Merit Medical, USA) was introduced through the base catheter into the genicular arteries supplying the pathological synovium (Fig. 2). Glycerol trinitrite (GTN) was injected through the microcatheter to optimize anterograde flow into the hypervascular synovium. A sports ice pack was placed on the skin surface of the knee corresponding to the area to be embolized. The hypothesis for the use of an icepack is that the reduced temperature will result in temporary vasoconstriction, minimizing non-target embolization to cutaneous arterial branches. The ice pack was left in situ for 15-min. Cone-beam-CT (Philips Allura FD20) was performed, injecting 6 ml of 100% iodinated contrast (Iomeron, Bracco, Italy) at 0.3 ml/s with a 6 s delay. This enabled confirmation of the hyperaemic target and identified contrast enhancement of adjacent skin, muscle, or bone, minimizing non-target embolization. Once a safe and effective microcatheter position was confirmed, the target vessels were embolized with 100–300 μm Embosphere particles (Merit Medical, USA) dilute in 20 ml (300 mg/ml) iodinated contrast (Iomeron, Bracco, Italy). Embolization was performed cautiously injecting 0.1–0.3 ml of embolic at a time using a 3-ml syringe. The aim of GAE in the osteoarthritic knee is to “prune” the abnormal vessels and maintain the larger native distal genicular branches (Fig. 2). The mean fluoroscopy duration was 14.29 min (SD = 9.58), and mean cumulative air kerma was 96 mGy (SD = 75). Patients recovered in the interventional radiology recovery unit for 4-h and were discharged home the same day. In order to minimize bias, data collection was completed by the radiology research nursing team, rather than the interventional radiologists performing the procedure. Technical success was defined as selective catheterization and embolization of the target genicular arteries. The minimum clinically important difference (MCID) for GAE has not been defined. Based on work by Roos, a MCID of 10 was used for KOOS scores, which is the upper limit of the recommended range [18].

**Fig. 3:**
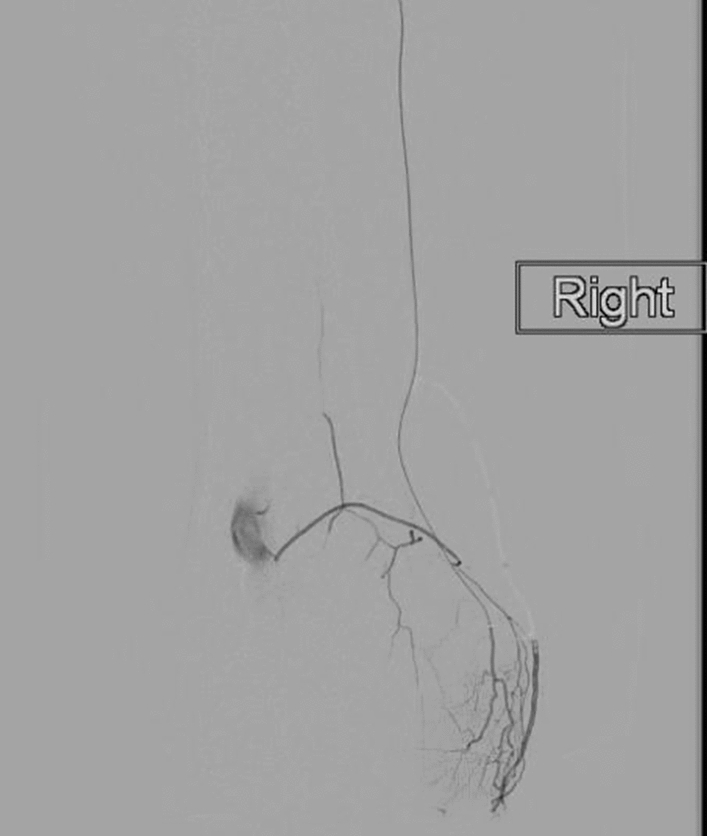
There is a rich anastomotic network between the genicular arteries. Contrast injected from the musculo-articular branch of the descending genicular artery reveals hyperaemic synovium within the medial knee compartment, and retrograde filling of the superior medical genicular, and popliteal arteries

### Statistical Analysis

The KOOS scores were summarized using descriptive statistics for each visit. Boxplots were also produced by visit. Adjusted means and differences from baseline (*n* = 32) along with 95% confidence intervals were derived. *p* values are presented for the differences between baseline and 6 weeks, 3 months (*n* = 32), and 1-year (*n* = 16). All applicable tests were two tailed using a 5% alpha significance level. WORMS inter-observer agreement was assessed by calculating intraclass correlation coefficients (ICC). The comparison of ratings was completed using a two-way mixed design, evaluating consistency, with 95% confidence intervals. Analyses were performed using MiniTab v.19.2020.01, Microsoft Excel v. 14.0.7015.1000, and SPSS Statistics v23.0.

## Results

Thirty-eight patients (median age = 60, range = 45–83) attended for GAE between June 2018 and January 2020 (Table 1). Eighteen patients with KL-grade 3, 17 with KL-grade 2, and 3 with KL-grade 1 were included. Despite cannulating all target genicular arteries, technical success was 84%, as six patients were not embolized: one due to significant cutaneous supply, and three because of anastomotic communication between the selected target genicular artery, and the popliteal artery, which might have resulted in distal non-target embolization (Fig. 3). Two further patients were not embolized due to a lack of hyperaemic target.

**Table 1:** Patient baseline characteristics

Age (years) median (range)	60 (45–83)
Female, *n* (%)	20 (53%)
BMI, median (range)	30 (20–48)
Analgesia (*n*)	
Paracetamol	12
NSAIDs	13
Opioids	5
Previous knee-specific treatments	
Physiotherapy	21
Previous intra-articular Injection	26
Previous arthroscopy	15
Acupuncture	2
PRP injection	1
Kellgren–Lawrence grade (*n*)	
1	3
2	17
3	18

*NSAID* non-steroidal anti-inflammatory drugs, *BMI* Body Mass Index, *PRP* platelet-rich plasma

**Fig. 4:**
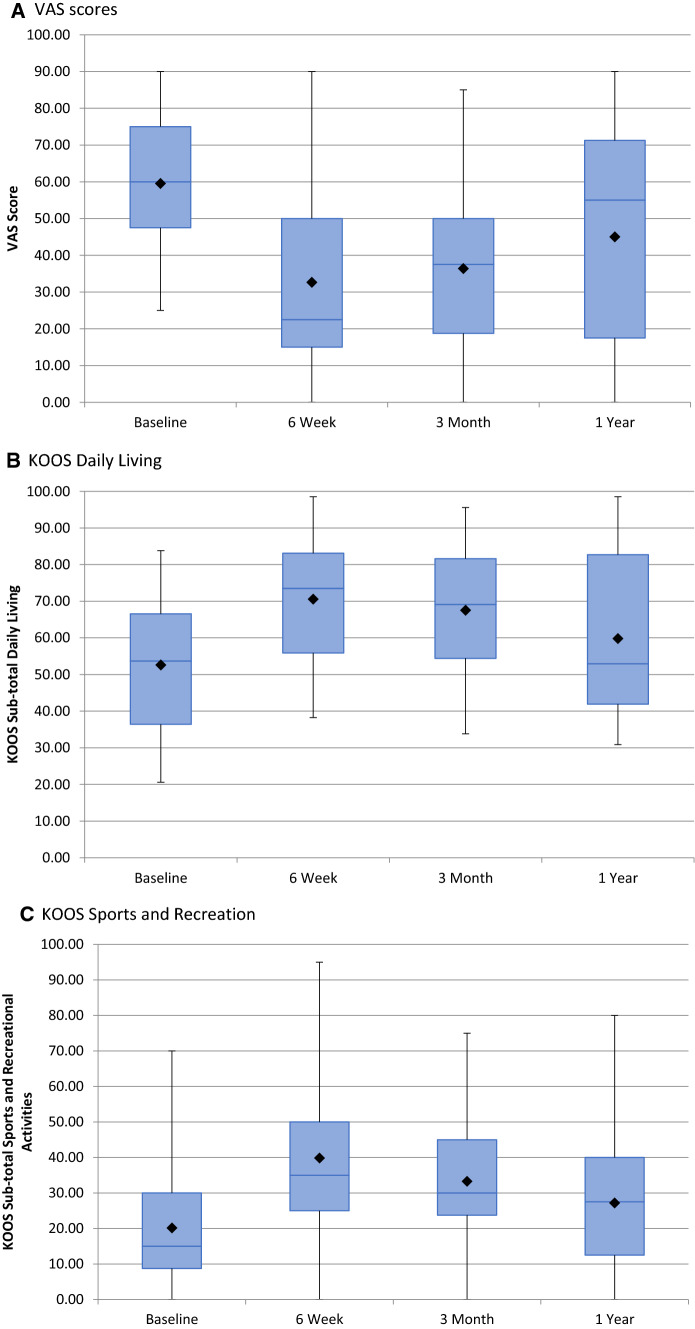

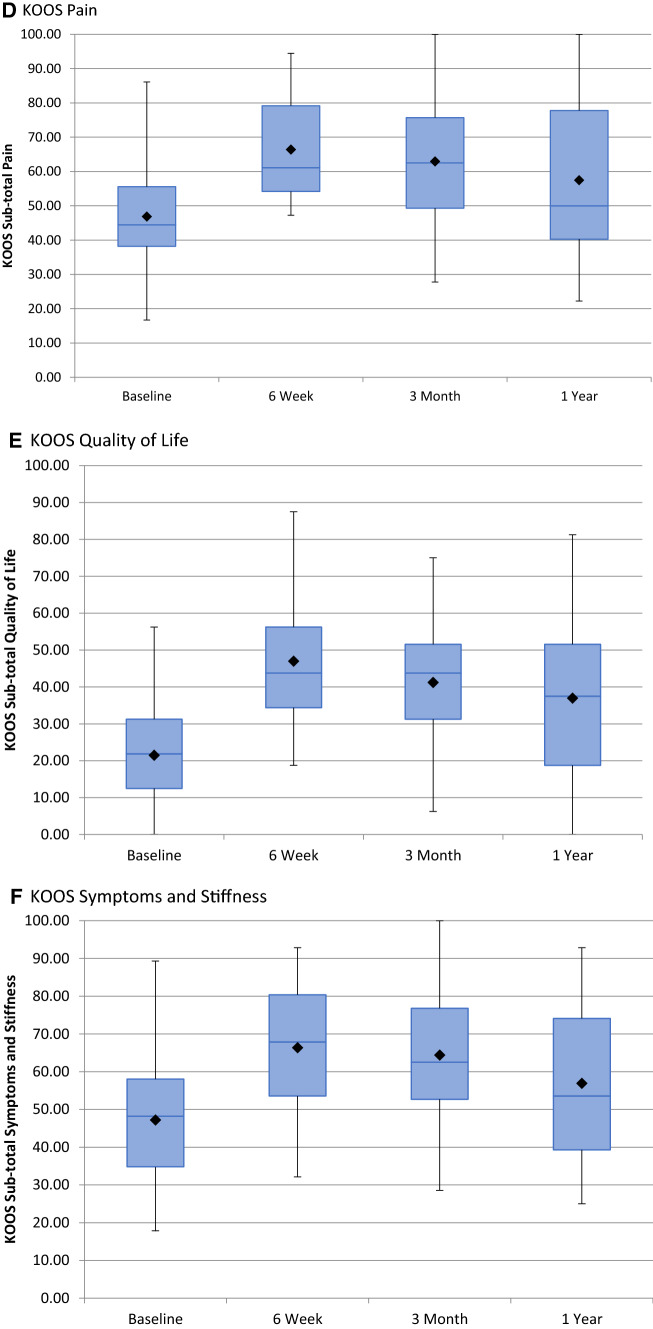
Boxplots depicting: VAS scores (**A**), KOOS subscale daily living (**B**), KOOS subscale sports and recreation (**C**), KOOS subscale pain (**D**), KOOS subscale quality of life (**E**), and KOOS subscale symptoms and stiffness (**F**)

The mean follow-up was 8 months (3–12 months). Mean VAS at baseline was 60 (SD = 20, 95% CI 53–66), reducing to 32 (SD = 25, 95% CI 24–42) at 6 weeks (*p* < 0.001), 36 (SD = 24, 95% CI 28–44) at 3 months (*p* < 0.001), and 45 (SD = 30, 95% CI 30–60) at 12 months (*p* < 0.05) (Fig. 4). KOOS subscales showed a statistically significant improvement from baseline to 6 weeks (*p* < 0.001), 3 months (*p* < 0.001), and 1-year (< 0.05) in all outcome measures except function in daily living, which revealed borderline significance at 12 months (*p* = 0.06) (Table 2, Fig. 4).

**Table 2:** Mean Knee Injury and Osteoarthritis Outcome Scores (KOOS) subscales

Assessment	Visit	Mean score	*p* value
Daily living	Baseline	52.62	
	6 weeks	70.59	< 0.01
	3 months	67.56	< 0.01
	12 months	59.83	0.06
Sports and recreational activities	Baseline	20.16	
	6 weeks	39.84	< 0.01
	3 months	33.28	< 0.01
	12 months	27.19	0.03
Pain	Baseline	45.46	
	6 weeks	66.40	< 0.01
	3 months	62.93	< 0.01
	12 months	57.47	0.02
Quality of life	Baseline	21.48	
	6 weeks	46.98	< 0.01
	3 months	41.21	< 0.01
	12 months	36.97	< 0.01
Symptoms and stiffness	Baseline	47.21	
	6 weeks	66.36	< 0.01
	3 months	64.40	< 0.01
	12 months	56.92	< 0.01

Interclass correlations indicated a good-to-excellent level of consistency between the two observers across total WORMS assessments [19]. ICCs were calculated between both, pre-treatment WORMS ratings (Average Measurement ICC = 0.952, 95% CI = 0.857–0.984, *F*(14, 14) = 20.89, *p* < 0.0001) and for post-treatment WORMS ratings (Average Measurement ICC = 0.933, 95% CI = 0.800–0.978, *F*(14, 14) = 14.93, *p* < 0.0001). WORMS analysis revealed a significant worsening of osteophytes, and bone attrition, with an improvement in synovitis (Table 3). ICCs were calculated to ensure consistency in the two observers’ evaluation of synovitis within the WORMs assessment. ICCs were found to be moderately reliable (Average Measurement ICC = 0.690, 95% CI = 0.078–0.896, *F*(14, 14) = 3.231, *p* < 0.0001 [19]. This facilitated the averaging of the two observer’s ratings and revealed significant improvement in synovitis from baseline to 1-year follow-up (*t*(14) = 2.39, *p* < 0.05). There were no cases of osteonecrosis.

**Table 3:** Whole-Organ Magnetic Resonance Imaging Score (WORMS) analysis

	Baseline (mean)	Baseline SD	1-year mean	1-year SD	*p* value
Cartilage	24	11.42	26.8	11.21	0.07
Marrow	4.53	4.47	5.2	4.57	0.10
Bone cysts	1	1.41	0.93	1.22	0.75
Bone attrition	1.13	1.19	1.73	1.44	0.03
Osteophytes	19.47	12.82	24.4	15.65	0.02
Menisci	2.73	2.22	2.87	2.23	0.33
Ligaments	0.13	0.35	0.13	0.35	1
Synovitis	1.73	0.88	1.13	0.51	0.01
Total	54.73	27.48	63.2	30.51	0.008

*SD* standard deviation

MCID results are shown in Table 4. MCID was met by 69%, 56%, 53%, 59%, and 69% of patients at 3 months for symptoms and stiffness, pain, daily living, sports and recreation, and quality of life, respectively.

**Table 4:** Minimum clinically important difference (MCID) for KOOS subscale at follow-up

KOOS outcome	6 weeks	3 months	1 year
	% of patients reaching MCID	% of patients reaching MCID	% of patients reaching MCID
Symptoms and stiffness	65	69	63
Pain	58	56	56
Daily living	65	53	50
Sports and recreational	61	59	44
Quality of life	81	69	63

Mean volume of embolic injected per patient was 1.6 ml (SD = 1.4). The mean number of genicular arteries embolized per patient was 1.3 (SD = 0.5). Despite technically successful GAE, two patients did not gain adequate symptomatic improvement at follow-up and subsequently underwent knee replacement surgery at a mean of 4 months following GAE. There were no adverse features reported by the orthopaedic surgeons performing these operations.

At baseline, 30 patients were taking regular analgesia for their knee pain (paracetamol = 12, NSAIDS = 13, opiates = 5). For the 32 patients undergoing GAE, 22 took regular analgesia (paracetamol = 8, NSAIDS = 10, opiates = 4). At 3-month follow-up, 15 patients were taking analgesia (paracetamol = 7, NSAIDS = 5, opiates = 3). For patients completing 1-year follow-up, 11 patients were taking regular analgesia (paracetamol = 4, NSAIDS = 4, Opiates = 3). No patients had any other knee-specific treatments during follow-up, except the two that went on to have knee arthroplasty.

27/38 (71%) of patients reported pain on injecting contrast and embolic into the target genicular arteries. Immediately post-embolization, these patients reported a significant improvement in this nociceptive response on repeat injection of contrast.

A pooled analysis of the patient satisfaction questionnaire results was performed (Table 5), defining a positive response as a given score of 1–5, and a negative response as 6–10 (reversed for question 7). This elucidated that 75% of responses were positive (*p* < 0.05) about GAE as a treatment for knee OA.

**Table 5:** Patient satisfaction questionnaire outcomes on acceptability of GAE

Question	Response scale	Mean response (standard deviation)
1. Did you feel anxious before the procedure?	1 (no)–10 (very)	2 (3)
2. Did you feel anxious during the procedure?	1 (no)–10 (very)	3 (3)
3. Did you feel any pain during the procedure?	1 (no)–10 (very)	4 (3)
4. Was the position of lying on your back uncomfortable?	1 (no)–10 (very)	3 (3)
5. Was the length of the procedure a problem?	1 (no)–10 (very)	3 (3)
6. Did you find it difficult to stay still for the procedure?	1 (no)–10 (very)	2 (2)
7. How did the procedure compare to your expectations?	1 (worse)–10 (better)	7 (4)
8. How did you find the procedure overall?	1 (not unpleasant)–10 (very unpleasant)	3 (3)
9. Would you have the procedure again?	1 (I wouldn’t mind)–10 (I would mind)	3 (3)

### Adverse Events

All adverse events were recorded prospectively in line with Cardiovascular and Interventional Radiological Society of Europe (CIRSE) Quality Assurance Document and Standards for Classification of Complications [20]. Four patients experienced mild self-limiting skin discoloration over the embolized territory as a result of non-target cutaneous embolization (12.5%). All cases completely resolved within 3 weeks (Grade-3). One patient experienced a small self-limiting groin haematoma (Grade 2). From the 16 patients completing 1-year follow-up, there were no cases of osteonecrosis of the knee joint detected on MRI.

## Discussion

Laboratory and animal experiments have identified the role of neo-angiogenesis in the pathophysiology of knee OA, and this has emerged as a viable embolization target. Existing human data, including the current work, have all reported a consistent signal that GAE is safe, technically achievable, and beneficial in reducing pain, and functional limitation secondary to mild to moderate knee OA at early follow-up [8–12]. The current analysis is the largest reported cohort with mild to moderate knee OA undergoing GAE with a permanent embolic. Most patients experienced an early significant improvement in pain and function at 6 weeks, which was maintained to 12 months. Bagla et al. [10] used 75 or 100 μm permanent Embozene particles (Boston Scientific, USA) to treat 20 patients with mild to moderate knee OA. They report the same data trend as the current study, with greater absolute improvement to 6 months. Differences in pain perception, disease severity, embolic size, suspension, and delivery require further study to elucidate the optimal patient selection and technique.

The most consistently reported adverse effect of GAE is non-target embolization to the overlying skin [21]. The use of cone-beam CT reveals the importance of recognizing cutaneous branches and the need to alter microcatheter position accordingly. Using cone-beam-CT, 100–300 μm particles, and applying an icepack to the skin over the area to be embolized resulted in 12% of patients experiencing non-target cutaneous embolization. This is substantially lower than comparable studies performing GAE with a permanent embolic, reporting non-target cutaneous embolization of 65% [10] and 57% [9]. Using 100–300 μm particles, we have had no cases of non-target embolization of the vascular supply to nerves, or bone.

WORMS analysis revealed a significant improvement in synovitis across all patients, with no significant cartilage loss. This finding was also reported by Okuno et al. [9], and is aligned with the hypothesis that synovial hypervascularity is an important factor in the pathological process of knee OA, which may be exploited by GAE to reduce pain and improve function [16, 22]. WORMS analysis also found a significant deterioration in osteophytes and bone attrition, not previously reported. Looking into the data, there were four patients with significant deterioration in osteophytes and bone attrition, with no significant difference in the remaining patients. These four patients had a median BMI of 35, which is significantly higher than the BMI of 25.2 for the cohort reported by Okuno et al. [9]. It is known that high BMI is a risk factor for the progression of knee OA [23]. The deterioration was seen throughout the knee joint in these four patients and was not limited to the compartments that were embolized. Despite significant worsening of osteophytes and bone attrition, these four patients had a mean improvement in KOOS pain from 40 at baseline to 58 at 12 months. Further investigation into the imaging following GAE is required.

An appreciation of the anastomoses between vessels, awareness of the embolization endpoint, and microcatheter experience is essential in order to perform safe GAE. Retrograde flow via genicular anastomoses may lead to non-target embolization (Fig. 3). We report three cases where there was significant retrograde flow into the popliteal artery, resulting in a presumed risk of non-target embolization. Whilst in other territories such as prostate artery embolization (PAE), it is safe and effective to coil embolize vessels to guard against non-target embolization; this practice has not been evaluated in GAE. There are two considerations underlying our rationale for not coiling vessels in GAE. Firstly, we are performing the procedure in relatively young patients (median age = 60); it is known that the genicular arteries are essential collateral vessels in peripheral vascular disease, and so there is a benefit in maintaining their supply [24]. Secondly, and perhaps most importantly, the genicular arteries provide the blood supply to the distal femur, proximal tibia, and patella. We therefore hypothesize that coil embolization combined with particulate embolization might disrupt the osseous blood supply, resulting in osteonecrosis. This will not only result in a poor outcome from the GAE procedure, but may also have a detrimental effect on future treatments such a joint replacement surgery.

The embolization endpoint is an important aspect of safe and effective GAE. Unlike other embolotherapies, the endpoint of GAE is not to occlude the proximal inflow vessel. The aim is to embolize the pathological hyperaemia, whilst maintaining the larger vascular supply to the bone (Fig. 3). This we describe as “pruning”, rather than blocking, and necessitates very careful instillation of embolic.

Patient-reported outcomes from satisfaction questionnaires were supportive of GAE as an acceptable procedure in patients with mild to moderate knee OA. Pain during the procedure returned the highest negative mean score from the satisfaction questionnaires. The pain experienced by these patients was from injecting contrast/embolic at pressure into the target genicular arteries. This was experienced by 27 patients, and was described as pain identical to their normal arthritis pain. Interestingly, when present, the pain desisted immediately following embolization. This phenomenon has been reported by other authors, and warrants further study [8, 9, 12].

The limitations of the current analysis are the small sample size, limited follow-up period, and the lack of experimental control group.

As with any intervention designed to benefit pain, the placebo effect must be considered. The correct exploration process should be translational from the laboratory, through animal models to humans, with safety and feasibility the initial priority. The complexity of measuring pain outcome following an intervention cannot be underestimated and should be a priority when designing future trials measuring response to GAE. As a result of this interim analysis, the GENESIS study will now follow patients to 2 years.

In conclusion, GAE of mild to moderate knee OA with a permanent microsphere is safe, and technically achievable, with potential efficacy at early follow-up. This work adds to the growing evidence that GAE has the potential to improve pain, function, and quality of life in patients with knee OA. Further study with a control group is justified.

## References

[1] Altman RD (2010). Early management of osteoarthritis. Am J Manag Care.

[2] Dieppe P, Lim K, Lohmander S (2011). Who should have knee joint replacement surgery for osteoarthritis?. Int J Rheum Dis.

[3] Canovas F, Dagneaux L (2018). Quality of life after total knee arthroplasty. Orthop Traumatol Surg Res.

[4] Mapp PI, Walsh DA (2012). Mechanisms and targets of angiogenesis and nerve growth in osteoarthritis. Nat Rev Rheumatol.

[5] Ashraf S, Mapp PI, Walsh DA (2011). Contributions of angiogenesis to inflammation, joint damage, and pain in a rat model of osteoarthritis. Arthritis Rheum.

[6] Ashraf S, Wibberley H, Mapp PI, Hill R, Wilson D, Walsh DA (2011). Increased vascular penetration and nerve growth in the meniscus: a potential source of pain in osteoarthritis. Ann Rheum Dis.

[7] Suri S, Gill SE, Massena de Camin S, Wilson D, McWilliams DF, Walsh DA (2007). Neurovascular invasion at the osteochondral junction and in osteophytes in osteoarthritis. Ann Rheum Dis.

[8] Okuno Y, Korchi AM, Shinjo T, Kato S (2015). Transcatheter arterial embolization as a treatment for medial knee pain in patients with mild to moderate osteoarthritis. Cardiovasc Interv Radiol.

[9] Okuno Y, Korchi AM, Shinjo T, Kato S, Kaneko T (2017). Midterm clinical outcomes and mr imaging changes after transcatheter arterial embolization as a treatment for mild to moderate radiographic knee osteoarthritis resistant to conservative treatment. J Vasc Interv Radiol.

[10] Bagla S, Piechowiak R, Hartman T, Orlando J, Del Gaizo D, Isaacson A (2019). Genicular artery embolization for the treatment of knee pain secondary to osteoarthritis. J Vasc Interv Radiol.

[11] Lee SH, Hwang JH, Kim DH, So YH, Park J, Cho SB (2019). Clinical outcomes of transcatheter arterial embolisation for chronic knee pain: mild-to-moderate versus severe knee osteoarthritis. Cardiovasc Interv Radiol.

[12] Landers S, Hely R, Page R, Maister N, Hely A, Harrison B (2020). Genicular artery embolization to improve pain and function in early-stage knee osteoarthritis-24-month pilot study results. J Vasc Interv Radiol.

[13] Roos EM, Toksvig-Larsen S (2003). Knee injury and Osteoarthritis Outcome Score (KOOS)—validation and comparison to the WOMAC in total knee replacement. Health Qual Life Outcomes.

[14] Jensen MP, Chen C, Brugger AM (2003). Interpretation of visual analog scale ratings and change scores: a reanalysis of two clinical trials of postoperative pain. J Pain.

[15] Hash TW, Maderazo AB, Haas SB, Saboeiro GR, Trost DW, Potter HG (2011). Magnetic resonance angiography in the management of recurrent hemarthrosis after total knee arthroplasty. J Arthroplasty.

[16] Korchi AM, Cengarle-Samak A, Okuno Y, Martel-Pelletier J, Pelletier JP, Boesen M (2019). Inflammation and hypervascularization in a large animal model of knee osteoarthritis: imaging with pathohistologic correlation. J Vasc Interv Radiol.

[17] Peterfy CG, Guermazi A, Zaim S, Tirman PFJ, Miaux Y, White D (2004). Whole-Organ Magnetic Resonance Imaging Score (WORMS) of the knee in osteoarthritis. Osteoarthr Cartil.

[18] Roos EM, Lohmander LS (2003). The knee Injury and Osteoarthritis Outcome Score (KOOS): from joint injury to osteoarthritis. Health Qual Life Outcomes.

[19] Koo TK, Li MY (2016). A guideline of selecting and reporting intraclass correlation coefficients for reliability research. J Chiropr Med.

[20] Filippiadis DK, Binkert C, Pellerin O, Hoffmann RT, Krajina A, Pereira PL. Cirse quality assurance document and standards for classification of complications: the cirse classification system. cardiovascular and interventional radiology. 2017 [cited 2020 Sep 14];40(8). 10.1007/s00270-017-1703-4. 10.1007/s00270-017-1703-4 28584945

[21] Weidner ZD, Hamilton WG, Smirniotopoulos J, Bagla S (2015). Recurrent hemarthrosis following knee arthroplasty treated with arterial embolization. J Arthroplasty.

[22] Scanzello CR, Umoh E, Pessler F, Diaz-Torne C, Miles T, Dicarlo E (2009). Local cytokine profiles in knee osteoarthritis: elevated synovial fluid interleukin-15 differentiates early from end-stage disease. Osteoarthr Cartil.

[23] Zheng H, Chen C (2015). Body mass index and risk of knee osteoarthritis: systematic review and meta-analysis of prospective studies. BMJ Open.

[24] Ziegler MA, Distasi MR, Bills RG, Miller SJ, Alloosh M, Murphy MP (2010). Marvels, mysteries, and misconceptions of vascular compensation to peripheral artery occlusion. Microcirculation.

