# Correction to: Genicular artEry embolizatioN in patiEnts with oSteoarthrItiS of the Knee (GENESIS) Using Permanent Microspheres: Interim Analysis

**DOI:** 10.1007/s00270-021-02849-7

**Published:** 2021-04-21

**Authors:** M. W. Little, M. Gibson, J. Briggs, A. Speirs, P. Yoong, T. Ariyanayagam, N. Davies, E. Tayton, S. Tavares, S. MacGill, C. McLaren, R. Harrison

**Affiliations:** 1 University Department of Radiology, Royal Berkshire NHS Foundation Trust, Reading, UK; 2 Department of Orthopaedics, Royal Berkshire NHS Foundation Trust, Reading, UK; 3 University of Reading, Reading, UK

## Correction to: Cardiovasc Intervent Radiol 10.1007/s00270-020-02764-3

One of the patients in the interim analysis was diagnosed with a popliteal deep vein thrombosis (DVT) 15 days after GAE in the same leg. The patient was treated with oral anticoagulation and made a full recovery. Whilst this is unlikely to be directly related to the GAE procedure, it highlights the risk of DVT in patients with knee OA and immobility. It is important to be aware of this when following up patients undergoing GAE for knee OA.

